# Arrangements of human telomere DNA quadruplex in physiologically relevant K^+^ solutions

**DOI:** 10.1093/nar/gku1274

**Published:** 2014-12-24

**Authors:** D. Renčiuk, I. Kejnovská, P. Školáková, K. Bednářová, J. Motlová, M. Vorlíčková

*Nucleic Acids Research*, 2009, Vol. 37, No. 19, 6625–6634. doi:10.1093/nar/gkp701

In the above article, the authors’ affiliations were erroneously omitted. The affiliation for all authors is: Institute of Biophysics, Academy of Sciences of the Czech Republic, v.v.i. Kralovopolska, 135 CZ - 612 65 Brno, Czech Republic.

The Publisher apologizes for this mistake.

